# G-Computation to Causal Mediation Analysis With Sequential Multiple Mediators—Investigating the Vulnerable Time Window of HBV Activity for the Mechanism of HCV Induced Hepatocellular Carcinoma

**DOI:** 10.3389/fpubh.2021.757942

**Published:** 2022-01-07

**Authors:** An-Shun Tai, Yen-Tsung Huang, Hwai-I Yang, Lauren V. Lan, Sheng-Hsuan Lin

**Affiliations:** ^1^Institute of Statistics, National Yang Ming Chiao Tung University, Hsinchu, Taiwan; ^2^Institute of Statistical Science, Academia Sinica, Taipei, Taiwan; ^3^Genomics Research Center, Academia Sinica, Taipei, Taiwan; ^4^Department of Biostatistics, Johns Hopkins University, Baltimore, MD, United States

**Keywords:** causal inference, mechanism investigation, mediation analysis, path-specific effect, multiple mediators

## Abstract

Regression-based approaches are widely used in causal mediation analysis. The presence of multiple mediators, however, increases the complexity and difficulty of mediation analysis. In such cases, regression-based approaches cannot efficiently address estimation issues. Hence, a flexible approach to mediation analysis is needed. Therefore, we developed a method for using g-computation algorithm to conduct causal mediation analysis in the presence of multiple ordered mediators. Compared to regression-based approaches, the proposed simulation-based approach increases flexibility in the choice of models and increases the range of the outcome scale. The Taiwanese Cohort Study dataset was used to evaluate the efficacy of the proposed approach for investigating the mediating role of early and late HBV viral load in the effect of HCV infection on hepatocellular carcinoma (HCC) in HBV seropositive patients (*n* = 2,878; HCV carrier *n* = 123). Our results indicated that early HBV viral load had a negative mediating role in HCV-induced HCC. Additionally, early exposure to a low HBV viral load affected HCC through a lag effect on HCC incidence [OR = 0.873, 95% CI = (0.853, 0.893)], and the effect of early exposure to a low HBV viral load on HCC incidence was slightly larger than that of a persistently low viral load on HCC incidence [OR = 0.918, 95% CI = (0.896, 0.941)].

## Introduction

Epidemiology studies and other health-related studies often investigate the overall effect of a certain risk factor or exposure on health-related outcomes. Confirmation of such effects facilitates further elucidation of possible biological mechanisms. Path analysis and mediation analysis are often used to investigate causal mechanisms because they can decompose these effects into several pathways according to the involvement of various mediators of interest (1). Mediation analysis aims to assess how exposure affects the outcome of interest through mediators and sheds deep insight into the underlying mechanism of the relationship between the exposure and outcome. Causal mediation analysis, a branch of mediation analysis, explicitly defines the causal effects of interest based on a counterfactual (potential) outcome model (2–4). The counterfactual model denotes the hypothetical outcome (here, it indicates the “counterfactual level” of a certain variable of interest) an individual would have, under a hypothetical condition when the same individual had received a particular intervention on previous variables. It is called “counterfactual” because this individual might not have received this intervention in real world. Since causal mediation analysis accounts for non-linearity of outcomes and interactions between exposure and mediator, it expands the use of mediation analysis to more general conditions (2, 5–7). Additionally, in scenarios involving a single fixed exposure and a single mediator, several techniques have been proposed to account for various outcome scales, including dichotomous variables (8), time-to-event variables (9–12), and many others (13, 14).

**Figure 1:**
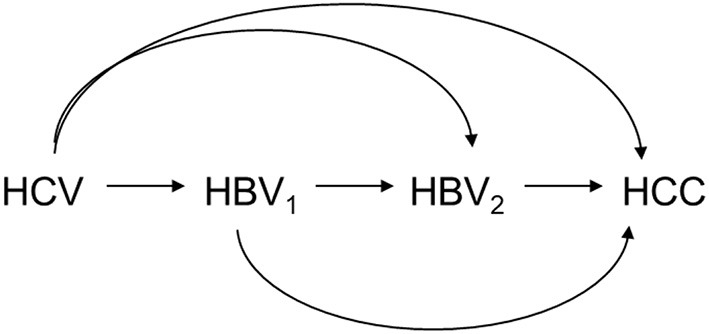
Causal relationship among HCV infection status (HCV), HBV viral load at baseline (HBV_1_), HBV viral load at follow-up (HBV_2_), and HCC status (HCC).

**Figure 2:**
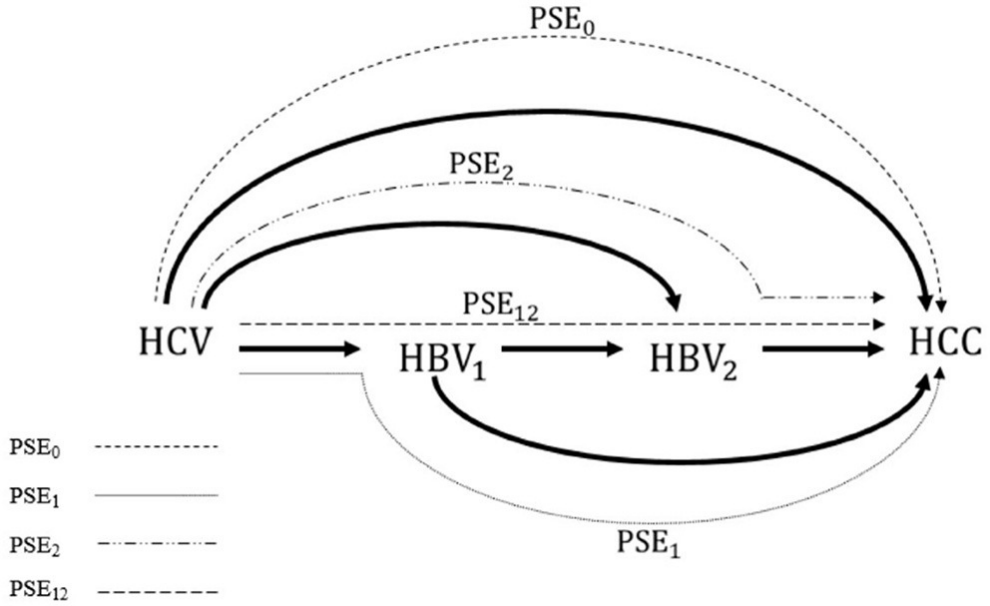
Four path-specific effects (PSEs), as well as four interventional PSEs, to be decomposed from the overall effect of HCV infection on the incidence of HCC. PSE_1_: the path through the HBV_1_ only; PSE_2_: the path through the HBV_2_ only, PSE_12_: the path through HBV_1_ which further impacts HBV_2_; and PSE_0_: the path not through HBV_1_ or HBV_2_. PSE, path-specific effect; HCV, hepatitis C virus; HBV, hepatitis B virus; HCC, hepatocellular carcinoma.

In multiple mediator settings, i.e., settings involving more than one mediator, however, mediation analysis is often challenging. One example is the extreme complexity of decomposing the effects of hepatitis C virus (HCV) infection on hepatocellular carcinoma (HCC) in the presence of hepatitis B virus (HBV) activity, which was the motivation for this study (15, 16). Figure 1 shows that the mediation analysis assumed causal relationships among HCV infection status, HBV viral load at baseline, HBV viral load at follow up, and HCC status. Baseline HBV viral load activity was used to represent the current status of HBV activity; baseline HCV infection status was used to represent relatively long-term HCV infection status. That is, HCV infection status was assumed to precede HBV viral load, which was considered a reasonable assumption. The role of HBV viral activity in this mechanism in HBV sero-positive patients at baseline and during follow up was investigated by using mediation analysis to decompose the effects into four paths (Figure 2). Effects in each of the four paths (i.e., the path-specific effects, PSEs) can be categorized as (1) paths only through change in early HBV viral load (PSE_1_); (2) paths only through change in late HBV viral load (PSE_2_); (3) paths through change in early HBV viral load that further impacts late HBV viral load (PSE_12_); and (4) paths not through change in early or late HBV viral load (PSE_0_). Decomposition of the overall effect into four PSEs facilitates understanding of the role of HBV viral activity and when the role of HBV viral activity is critical. These data can then be used to reduce the HCC incidence in patients with dual virus infection.

Before conducting mediation analysis in this case, the two settings must be differentiated according to the relationships between mediators. In the first setting, mediators are independent of each other conditioned on all previous covariates, including baseline confounders and the exposure. In this setting, which is also referred to as “parallel” or “non-ordered” multiple mediators, the motivating example is rational only if early HBV viral load does not affect late HBV viral load. The standard causal mediation analysis framework for a single mediator is easily extended to this setting by performing a sequential mediation analysis of each mediator. Notably, methods have also been developed for simultaneous analysis of parallel mediators (17, 18). Apparently, however, the above parallel setting does not fit our motivating example since early HBV viral activity would surely affect viral activity at follow up. In the case of early HBV viral activity, the alternative setting, “ordered” or “sequential” multiple mediators, is reasonable. Unfortunately, effect decomposition in this setting is infeasible since some PSEs cannot be identified by empirical data without additional strong assumptions (15, 19–21). For example, to identify full PSEs in the presence of two ordered mediators, the assumption of independence between two counterfactuals of the mediator is proposed for identification (21). This independence assumption is extremely strong and unrealistic. Without further assumptions, only partial effect decomposition, which evaluates the cumulative PSEs, can be achieved.

Specifically, only PSE_2_, PSE_0_, and the sum of PSE_1_ and PSE_12_ are identifiable. However, PSE_1_ and PSE_12_ cannot be further distinguished, even without time-varying and unmeasured baseline confounders. Two strategies for resolving this problem are possible. First, the overall effect can be decomposed into the three components described above (16, 22, 23). We can further pool all ordered mediators as a single mediator, and decompose the total effect into effect either through or not through this pooled mediator (18, 20). The second approach is to measure the upper and lower bounds of PSE through sensitivity analysis under causal framework (24, 25). However, point estimate of PSE still cannot be obtained through this method (21). Previously, Lin and VanderWeele proposed an interventional approach to estimate analogs of PSEs under no-unmeasured-confounding assumptions with a regression-based approach (26). The concepts of the interventional approach and PSE were also adopted by VanderWeele, Vansteelandt, and Robins (20) for mediation analysis with a single mediator in the presence of an exposure-induced mediator-outcome confounder. Note that their work only derives the direct effect, the sum of two PSEs passed through the mediator, and the indirect effect, the sum of two PSEs without passing through the mediator. Meanwhile, Vansteelandt and Daniel also proposed a new interventional approach, which has no assumption of structure among mediators, for deriving PSEs (27), but different from Lin and VanderWeele's method, they still cannot distinguish PSE_12_ from the other PSEs. A limitation of Lin and VanderWeele's method is that the link function of outcome model has to be linear or log-linear, and that it cannot be adapted for a non-linear or generalized linear models. Moreover, unlike the analysis of overall effect, the analytical solutions for all PSEs estimates vary substantially in different models even when the linear function of outcome model is linear or log-linear. Therefore, the software of the regression-based approach can only be applied to few model choices.

To remedy this research gap, we adopted the simulation-based approach based on g-computation algorithm to provide a flexible computational algorithm for the estimation of causal mediation analysis. g-computation algorithm was first introduced by Robins in 1986 to estimate the causal effect of a time-varying exposure in the presence of time-varying confounders that are affected by exposure (3). Recently, the simulation-based approach has been widely used for standard causal mediation analysis (27–34). These methods usually involve using maximum likelihood estimation (MLE) to fit a set of parametric models and then using g-computation algorithm and bootstrapping methods to generate point and interval estimates, respectively. This simulation-based approach provides the flexibility to choose models and variables without considering an analytic form. This approach also obtains more stable and efficient estimates compared to weighted approach (14, 31, 35). Therefore, simulation-based approach is useful for investigating mechanisms when the outcome variable does not fit the requirements of a linear regression model. Therefore, this study used this approach to develop a method of performing mediation analysis in scenarios involving two ordered multiple mediators. The proposed method was then used investigate the mechanisms through which HCV induces HCC through HBV activity.

## Materials and Methods

### Data Description of the REVEAL-HBV Study

This study was motivated by the Risk Evaluation of Viral Load Elevation and Associated Liver Disease/Cancer–Hepatitis B Virus (REVEAL-HBV) study (36). The details of the REVEAL-HBV study design and participant enrollment have been illustrated in literatures (36–39). 23,820 Taiwanese residents aged 30–65 years were recruited from 1991 to 1992. Among the participants, 2,878 were HBV-positive, of which 188 developed HCC during the follow-up period. Written informed consent for interview questionnaires, health examinations, biospecimen collection, and data linkage of health status with death certification profiles and National Cancer Registry were obtained. Blood samples collected at enrollment were examined for seromarkers and viral load of HBV and HCV. Newly diagnosed HCC was recorded using computerized data linkage with National Cancer Registry and death certification systems.

### Notation, Definition, and Effect Decomposition for Dichotomous Outcome

**Figure 3:**
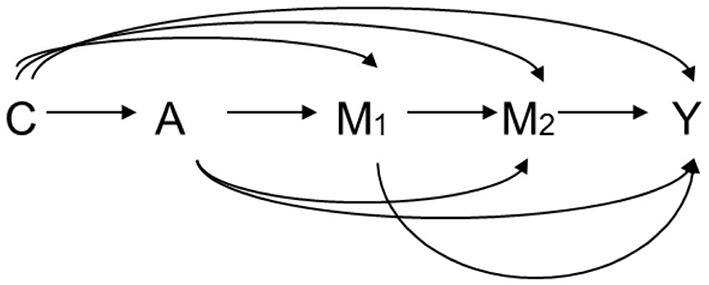
Relation among exposure A, two ordering mediators M_1_ and M_2_, outcome Y, and covariates C. A: exposure, M_1_: the first mediator, M_2_: the second mediator, Y: outcome, C: covariates.

**Figure 4:**
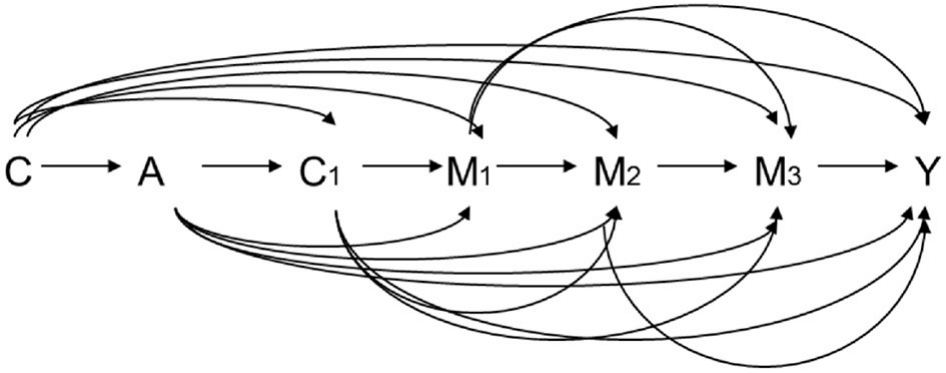
Relation among exposure A, three ordering mediators M_1_, M_2_, and M_3_, outcome Y, baseline covariates C, and time-varying mediator-outcome confounder C_1_. A: exposure, M_1_: the first mediator, M_2_: the second mediator, Y: outcome, C: baseline covariates, C_1_: time-varying mediator-outcome confounder.

Let A denote the exposure, Y a dichotomous outcome, M_1_ the first mediator, M_2_ the second mediator, and C a set of baseline covariates. For example, A is HCV infection status, Y is an HCC event before the end of follow up, M_1_ is early HBV viral load, and M_2_ is late HBV viral load. Let A = 1 and A = 0 denote two hypothetical levels of exposure: HCV infection and non-infection, respectively. Figure 3 graphically illustrates the causal relationships among A, Y, M_1_, M_2_, and C based on substantive prior knowledge. Figure 4 is the case of more than two mediators as well as time-varying mediator-outcome confounders, which are affected by exposure. For simplicity, however, we assume the absence of time-varying confounders, and we assume the presence of only two ordered mediators of interest.

Counterfactual outcome models are used to define four PSEs corresponding the four paths in Figure 2 based on causal theory (2–6, 19, 40). For the individual i, Y_i_(a) denotes the counterfactual level of Y_i_ if this individual had received an intervention on exposure A as level a. Similarly, M_2i_(a, m_1_) denotes the counterfactual level of M_2i_ if this individual had received an intervention on exposure A as level a and on the first mediator M_1i_ as level m_1_. Here, the notation can be simplified by removing the subscript i.

For a dichotomous outcome, the total effect may be expressed on risk difference (RD), risk ratio (RR), or odds ratio (OR) scale. Although software used to perform simulation-based approaches provides the results of all scales, the OR scale is used throughout this discussion since OR is the most frequently used scale for dichotomous outcomes. The total effect in OR scale, OR_TE_(1,0), is defined as Odds(Y(1))/Odds(Y(0)), where Odds(B) is defined as Pr(B = 1)/Pr(B = 0) for any dichotomous variable B [e.g. Y, Y(1), or Y(0)]. The definitions of RD and RR scales are detailed in Appendix A.

When investigating a mechanism with two mediators M_1_ and M_2_ of interest, the total effect (OR_TE_) can be decomposed into four PSEs: path not through M_1_ or M_2_; path through M_1_ only; path through M_2_ only; and path through M_1_ and then through M_2_; these four PSEs are expressed in OR scale as OR_PSE0_, OR_PSE1_, OR_PSE2_, and OR_PSE12_, respectively, and are defined as follows:


(1) $$\begin{array}{l} \;{\text{OR}}_{\text{PSE}0}=\Phi \left(1,0,0,0\right)/\Phi \left(0,0,0,0\right)\\ \;{\text{OR}}_{\text{PSE}1}=\Phi \left(1,1,0,0\right)/\Phi \left(1,0,0,0\right)\\ \;{\text{OR}}_{\text{PSE}2}=\Phi \left(1,1,1,0\right)/\Phi \left(1,1,0,0\right)\\ {\text{OR}}_{\text{PSE}12}=\Phi \left(1,1,1,1\right)/\Phi \left(1,1,1,0\right) \end{array}$$

where Φ(a_1_,a_2_,a_3_,a_4_) is defined as Odds(Y(a_1_,M_1_(a_2_),M_2_(a_3_,M_1_(a_4_)))). Here, Y(a_1_,M_1_(a_2_),M_2_(a_3_,M_1_(a_4_))) denotes the counterfactual value of outcome Y if the exposure is set to a_1_, the first mediator is set to M_1_(a_2_), and the second mediator is set to M_2_(a_3_,M_1_(a_4_)) (or the counterfactual value of M_2_ if exposure is set to a_3_ and first mediator is set to M_1_(a_4_)). The OR_TE_ is the product of four PSEs in OR scale, which can be expressed as


(2) $$\begin{array}{l} {\text{OR}}_{\text{TE}}=OR_{\text{PSE}0}\times {\text{OR}}_{\text{PSE}1}\times {\text{OR}}_{\text{PSE}2}\times {\text{OR}}_{\text{PSE}12} \end{array}$$

While Equation (1) gives a definition of four PSEs decomposed from TE, the decomposition of TE is not unique. For example, OR_PSE0_ = Φ(1,1,1,1)/Φ(0,1,1,1), OR_PSE1_ = Φ(0,1,1,1)/Φ(0,0,1,1), OR_PSE2_ = Φ(0,0,1,1)/Φ(0,0,0,1), and OR_PSE12_ = Φ(0,0,0,1)/Φ(0,0,0,0) are alternative decomposition of TE. For two sequential mediators, 24 possible decompositions have been provided in the previous study (21). This work primarily focuses on the decomposition type defined in Equation (1). The following identification and estimation are valid no matter which decomposition is used.

### Interventional Approach to Identification

The Φ(a_1_,a_2_,a_3_,a_4_) can be non-parametrically identified only when a_2_ is equal to a_4_. Consequently, only OR_PSE0_, OR_PSE2_ and the sum of OR_PSE1_ and OR_PSE12_ are identified by empirical data. Here, we introduce an interventional approach: instead of defining the four paths as four traditional PSEs, the four paths are defined as four interventional path-specific effects (iPSEs). In an earlier work, these paths were referred to as randomly interventional analogs of PSEs (26). The advantage of the interventional approach is that all iPSEs can be non-parametrically identified under the assumption of no unmeasured confounding factors. (26). In OR scale, the paths are denoted OR_iPSE0_, OR_iPSE1_, OR_iPSE2_, and OR_iPSE12_ and are defined as follows:


(3) $$\begin{array}{l} \;{\text{OR}}_{\text{iPSE}0}=\text{Ψ}\left(1,0,0,0\right)/\text{Ψ}\left(0,0,0,0\right)\\ \;{\text{OR}}_{\text{iPSE}1}=\text{Ψ}\left(1,1,0,0\right)/\text{Ψ}\left(1,0,0,0\right)\\ \;{\text{OR}}_{\text{iPSE}2}=\text{Ψ}\left(1,1,1,0\right)/\text{Ψ}\left(1,1,0,0\right)\\ {\text{OR}}_{\text{iPSE}12}=\text{Ψ}\left(1,1,1,1\right)/\text{Ψ}\left(1,1,1,0\right) \end{array}$$

where Ψ(a_1_,a_2_,a_3_,a_4_) is defined as Odds(Y(a_1_,G_1_(a_2_),G_2_(a_3_,G_1_(a_4_)))). Here, we set the exposure as a_1_, the first mediator as G_1_(a_2_), and the second mediator as G_2_(a_3_,G_1_(a_4_)). For any value of a and m, G_1_(a) is the random draw of M_1_(a), and G_2_(a,m_1_) is the random draw of M_2_(a,m_1_). In this setting, Y(a_1_,G_1_(a_2_),G_2_(a_3_,G_1_(a_4_))) denotes the counterfactual value of outcome Y . Consequently, G_2_(a_3_,G_1_(a_4_)) is the random draw of M_2_(a_3_,G_1_(a_4_)) while G_1_(a_4_) is the random draw of M_1_(a_4_). As in the conventional definition, the interventional definition for each path replaces the counterfactual level of each mediator with its random draw. We further define the product of four OR_iPSE_ as the interventional total effect (iTE), which can be expressed in OR scale as the following equation:


(4) $$\begin{array}{l} {\text{OR}}_{\text{iTE}}={\text{OR}}_{\text{iPSE}0}\times {\text{OR}}_{\text{iPSE}1}\times {\text{OR}}_{\text{iPSE}2}\times {\text{OR}}_{\text{iPSE}12} \end{array}$$

The OR_iTE_ are very similar to the standard OR_TE_ but not identical (14, 35). Therefore, as in the effect decomposition of OR_TE_, the interventional decomposition can be viewed as its analog. The interpretations obtained when using iTE and iPSE, which are defined according to the stochastic interventions, differ from those of TE and PSE. These interpretations might be the best interpretations for a mechanism investigation as only the upper and lower bounds on PSE can be identified by empirical data even without time-varying confounders. Since iPSEs are PSEs analogs, iPSEs can still capture pathways. For example, OR_iPSE12_ is non-zero only under the following conditions: (1) the change in the exposure affects the distribution of the first mediator; (2) the change in the first mediator affects the distribution of the second mediator; and (3) the change in the second mediator affects the distribution of the outcome. In extremely pathological settings, iPSEs may fail to represent the effects obtained by traditional PSEs. One example is the case of no overlap among individuals in whom the exposure affects the first mediator, individuals in whom the first mediator affects the second mediator, and individuals in whom the second mediator affects the outcome. In this case, OR_iPSE12_ is non-zero while OR_PSE12_ is under null. In contrast, in the case of complete overlap among all of these individuals (i.e., in the case of complete overlap among individuals in whom the exposure affects the first mediator, individuals in whom the first mediator affects the second mediator, and individuals in whom the second mediator affects the outcome) OR_iPSE12_ is biased toward null. Further research on this topic in needed to elucidate the de*via*tion between PSE and its interventional version in different scenarios and to extend the applications of our method.

To identify Ψ(a_1_,a_2_,a_3_,a_4_) and to identify OR_iPSE_ and OR_iTE_, four no-unmeasured-confounding assumptions are required:

Assumption (1) no-unmeasured-confounding between the relationships of exposure and outcome

Assumption (2) no-unmeasured-confounding between the relationships of mediators and outcome;

Assumption (3) no-unmeasured-confounding between the relationships of exposure and mediators;

Assumption (4) no-unmeasured-confounding between the relationships of two mediators.

Assumptions (1) to (4) are essentially used to avoid confounding bias in estimating iPSEs. It is worthy to note that a further cross-world assumption of no exposure-induced mediator-outcome confounder is commonly made in the conventional approaches of mediation analysis (9, 15, 21) but is unnecessary to the interventional approach. Using random draw permits that iPSEs are identifiable even when an exposure-induced mediator-outcome confounder presents. Here, we consider the case without an exposure-induced mediator-outcome confounder for identification. The identification result can be straightforwardly extended to the case where mediator-outcome confounders are affected by exposure directly. Under assumptions (1) to (4), OR_iPSE_ and OR_iTE_ are identified as follows:


(5) $$\begin{array}{l} \;{\text{OR}}_{\text{iTE}}=V\left(1,1,1,1\right)/V\left(0,0,0,0\right)\\ \;{\text{OR}}_{\text{iPSE}0}=V\left(1,0,0,0\right)/V\left(0,0,0,0\right)\\ \;{\text{OR}}_{\text{iPSE}1}=V\left(1,1,0,0\right)/V\left(1,0,0,0\right)\\ \;{\text{OR}}_{\text{iPSE}2}=V\left(1,1,1,0\right)/V\left(1,1,0,0\right)\\ {\text{OR}}_{\text{iPSE}12}=V\left(1,1,1,1\right)/V\left(1,1,1,0\right) \end{array}$$

where *V*(*a*_1_, *a*_2_, *a*_3_, *a*_4_) is defined as $\frac{Q\left(a_{1},a_{2},a_{3},a_{4}\right)}{\left(1-Q\left(a_{1},a_{2},a_{3},a_{4}\right)\;\right)}$

and


(6.1) $$\begin{array}{l} Q\left(a_{1},a_{2},a_{3},a_{4}\right)=\\ \sum_{c}\sum_{m_{2},m_{1}}\mathrm{Pr}\left[Y=1|C=c,\;A=a_{1},M_{1}=m_{1},M_{2}=m_{2}\right]\\ \mathrm{Pr}\left(M_{1}=m_{1}|C=c,\;A=a_{2}\right)\times \sum m_{1}^{{}^{\prime }}\\ \mathrm{Pr}\left(M_{2}=m_{2}|C=c,A=\;a_{3},M_{1}=m_{1}^{\prime }\right)\\ \mathrm{Pr}\left(M_{1}=m_{1}^{\prime }|C=c,A=\;a_{4}\right)\mathrm{Pr}\left(C=c\;\right) \end{array}$$

If both M_1_ and M_2_ are continuous variables, (6.1) are replaced by integrals (6.2):


(6.2) $$\begin{array}{l} Q\left(a_{1},a_{2},a_{3},a_{4}\right)=\\ \int_{c}\int_{m_{2},m_{1}}\{\mathrm{Pr}\left[Y=1|C=c,A=a_{1},M_{1}=m_{1},M_{2}=m_{2}\right]\\ dF_{M_{1}|C,A}\left(M_{1}=m_{1}|C=c,\;A=a_{2}\right)\}\times \\ \int_{m_{1}^{\prime }}\{dF_{M_{2}|C,A,M_{1}}\left(M_{2}=m_{2}|C=c,\;A=a_{3},M_{1}=m_{1}^{\prime }\right)\\ dF_{M_{1}|C,A}\left(M_{1}=m_{1}^{\prime }|C=c,\;A=a_{4}\right)\}dF_{C}\left(c\right) \end{array}$$

A previous work provide the proof for a generalized case in the presence of time-varying confounders (26). Appendix A defines iPSEs in RD and RR scales.

A logistic regression or other non-linear model can be used to estimate the conditional probability of outcome. Without assuming a rare disease (conditional probability of outcome < 10%), Q(a_1_,a_2_,a_3_,a_4_) cannot be adequately approximated by a closed form. Consequently, a regression-based method is inapplicable, which was our motivation for developing the proposed simulation-based approach. In the simulation-based approach, the g-computation algorithm for iPSE is used for point estimation, and bootstrapping procedures are used for interval estimation. Since it does not consider the existence of the analytic form for all estimations, the simulation-based approach provides flexibility in the selection of statistical models.

### Simulation-Based Approach for Estimation

In the proposed simulation-based approach, we use g-computation algorithm for iPSE point estimation and bootstrapping procedures for interval estimation. First, we build parametric models for the outcome and two mediators. For example, if two mediators are continuous variables and the outcome is a binary variable, three regression models are built:


(7.1) $$\begin{array}{l} logit\left(\mathrm{Pr}\left(Y=1|A=a,\;M_{1}=m_{1},\;M_{2}=m_{2},\;C=c\right)\right)\\ =\theta_{0}+\theta_{1}a+\theta_{2}m_{1}+\theta_{3}m_{2}+\theta_{c}c \end{array}$$

(7.2) $$\begin{array}{l} E\left(M_{2}|A=a,\;M_{1}=m_{1},\;C=c\right)\\ =\beta_{0}+\beta_{1}a+\beta_{2}m_{1}+\beta_{c}c \end{array}$$

(7.3) $$\begin{array}{l} E\left(M_{1}|A=a,\;C=c\right)=\gamma_{0}+\gamma_{1}a+\gamma_{c}c \end{array}$$

The simulation-based approach allows for flexible selection of statistical models. Without considering the existence of the analytic form for all estimation, we can use any link function such as complementary log or probit function. Quadratic term or even log transformation or exposure and an interaction term between the exposure and the first mediator in model (7.1) can be included:


(8) $$\begin{array}{l} clog\left(-log\left(1-Pr\left(Y=1|A=a,\;M_{1}=m_{1},\;M_{2}=m_{2},\;C=c\right)\right)\right)\\ \;=\theta_{0}+\theta_{1}a+\theta_{1s}a^{2}+\theta_{1l}\log\left(a\right)\\ \;+\theta_{2}m_{1}+\theta_{12}am_{1}+\theta_{3}m_{2}+\theta_{c}c \end{array}$$

After building parametric models for two mediators and outcome, we fit these models and obtain MLEs for all parameters. Based on all MLEs, we simulate the point estimations Q(1,1,1,1), Q(1,1,1,0), Q(1,1,0,0), Q(1,0,0,0), and Q(0,0,0,0) based on equation (6), as well as four OR_iPSE_ and OR_iTE_ based on the definition in (5). We generate confidence intervals by bootstrapping for the PSE inference as follows.

(step 1) Construct a regression model for conditional distribution *M*_1_, *M*_2_, *and Y*.
  (1a) Construct a regression model for *M*_1_ on *A* and all confounders.
  (1b) Construct a regression model for *M*_2_ on *M*_1_, *A* and all confounders.
  (1c) Construct a regression model for *Y* on *M*_2_, *M*_1_, *A* and all confounders.

For example, we can construct models using the following procedure as models (7.1)–(7.3):


$$\begin{array}{l} M_{1}=\theta_{1,0}+\theta_{1,a}A+{\tilde{\theta }}_{1,c}\tilde{C}+\varepsilon_{1} \end{array}$$

$$\begin{array}{l} M_{2}=\theta_{2,0}+\theta_{2,a}A+\theta_{2,1}M_{1}+{\tilde{\theta }}_{2,c}\tilde{C}+\varepsilon_{2} \end{array}$$

$$\begin{array}{l} u_{y}={\left[1+\exp\left(-\left(\theta_{y,0}+\theta_{y,a}A+\theta_{y,1}M_{1}+\theta_{y,2}M_{2}+{\tilde{\theta }}_{y,c}\tilde{C}\right)\right)\right]}^{-1} \end{array}$$

$$\begin{array}{l} \tilde{C}={\left(C_{1},C_{2},\ldots ,C_{n_{c}}\right)}^{T} \end{array}$$

$$\begin{array}{l} {\tilde{\theta }}_{1,c}=\left(\theta_{1,c_{1}},\;\theta_{1,c_{2}},\ldots ,\;\theta_{1,c_{n_{c}}}\right) \end{array}$$

$$\begin{array}{l} {\tilde{\theta }}_{2,c}=\left(\theta_{2,c_{1}},\;\theta_{2,c_{2}},\ldots ,\;\theta_{2,c_{n_{c}}}\right) \end{array}$$

$$\begin{array}{l} {\tilde{\theta }}_{y,c}=\left(\theta_{y,c_{1}},\;\theta_{y,c_{2}},\ldots ,\theta_{y,c_{n_{c}}}\right) \end{array}$$

$$\begin{array}{l} Y\sim \;Bernoulli\left(\mu_{y}\right),\varepsilon_{1}\sim \;normal\left(0,\;\sigma_{1}^{2}\right),\varepsilon_{2}\sim \;normal\left(0,\;\sigma_{2}^{2}\right) \end{array}$$

(step 2) Fit models with real data to obtain MLE for all parameters, i.e.

$$\begin{array}{l} {\hat{\theta }}_{1,0},{\hat{\theta }}_{1,a},{\hat{\tilde{\theta }}}_{1,c},\;{\hat{\theta }}_{2,0},{\hat{\theta }}_{2,a},{\hat{\theta }}_{2,1},{\hat{\tilde{\theta }}}_{2,c},\;{\hat{\theta }}_{y,0},{\hat{\theta }}_{y,a},{\hat{\theta }}_{y,1},{\hat{\theta }}_{y,2},{\hat{\tilde{\theta }}}_{y,c},\;{\hat{\sigma }}_{1}^{2},\\ \;and\;{\hat{\sigma }}_{2}^{2}. \end{array}$$

(step 3) Conduct g-computation algorithm using MLE and bootstrap.
  (3a) Randomly sample the confounders $\tilde{C}$ with replacement and intervene the exposure *A* as 1. Use models built in Step 1 and MLEs in Step 2 to generate *M*_1_ [denoted as *G*_1_(1)].
  (3b) Randomly sample the confounders $\tilde{C}$ with replacement, and intervene the exposure *A* as 0. Use models built in Step 1 and MLEs in Step 2 to generate *M*_1_ [denoted as *G*_1_(0)].
  (3c) Randomly sample the confounders $\tilde{C}$, *G*_1_(1) with replacement, and intervene the exposure *A* as 1 and *M*_1_ as *G*_1_(1). Use models built in Step 1 and the MLEs in Step 2 to generate *M*_2_ [denoted as *G*_2_(1, *G*_1_(1))].
  (3d) Randomly sample the confounders $\tilde{C}$, *G*_1_(0) with replacement, and intervene the exposure *A* as 1 and *M*_1_ as *G*_1_(0). Then use models from Step 1 and MLEs in Step 2 to generate *M*_2_ [denoted as *G*_2_(1, *G*_1_(0))].
  (3e) Randomly sample the confounders $\tilde{C}$, *G*_1_(0) with replacement, and intervene the exposure *A* as 0 and *M*_1_ as *G*_1_(0). Use models constructed in Step 1 and MLEs from Step 2 to generate *M*_2_ [denoted as *G*_2_(0, *G*_1_(0))].
  (3f) Randomly sample the confounders $\tilde{C}$, *G*_1_(1), *G*_2_(1, *G*_1_(1)) with replacement, and intervene the exposure *A* as 1, *M*_1_ as *G*_1_(1), and *M*_2_ as *G*_2_(1, *G*_1_(1)). Use models built in Step 1 and MLEs from Step 2 to generate *Y* [denoted as *Y*(1, *G*_1_(1), *G*_2_(1, *G*_1_(1)))].
  (3g) Randomly sample the confounders $\tilde{C}$, *G*_1_(1), *G*_2_(1, *G*_1_(0)) with replacement, and intervene the exposure *A* as 1, *M*_1_ as *G*_1_(1), and *M*_2_ as *G*_2_(1, *G*_1_(0)). Use models built in Step 1 and MLEs from Step 2 to generate *Y* [denoted as *Y*(1, *G*_1_(1), *G*_2_(1, *G*_1_(0)))].
  (3h) Randomly sample the confounders $\tilde{C}$, *G*_1_(1), *M*_2_(0, *G*_1_(0)) with replacement, and intervene the exposure *A* as 1, *M*_1_ as *G*_1_(1), and *M*_2_ as *G*_2_(0, *G*_1_(0)). Use models built in Step 1 and MLEs from Step 2 to generate *Y* [denoted as *Y*(1, *G*_1_(1), *G*_2_(0, *G*_1_(0)))].
  (3i) Randomly sample the confounders $\tilde{C}$, *G*_1_(0), *G*_2_(0, *G*_1_(0)) with replacement, and intervene the exposure *A* as 1, *M*_1_ as *G*_1_(0), and *M*_2_ as *G*_2_(0, *G*_1_(0)). Use models built in Step 1 and MLEs from Step 2 to generate *Y* [denoted as *Y*(1, *G*_1_(0), *G*_2_(0, *G*_1_(0)))].
  (3j) Randomly sample the confounders $\tilde{C}$, *G*_1_(0), *G*_2_(0, *G*_1_(0)) with replacement, and intervene the exposure *A* as 0, *M*_1_ as *G*_1_(0), and *M*_2_ as *G*_2_(0, *G*_1_(0)). Use models built in Step 1 and MLEs from Step 2 to generate *Y* [denoted as *Y*(0, *G*_1_(0), *G*_2_(0, *G*_1_(0)))].
  (3k) Compute the means *Y*(*a*_1_, *G*_1_(*a*_2_), *G*_2_(*a*_3_, *G*_1_(*a*_4_))), *for i* = 1, 2, 3, 4, *and a*_*i*_ ∈ {0, 1}, which is the g-computation algorithm approximation estimation of *Q*(*a*_1_, *a*_2_, *a*_3_, *a*_4_,). Based on formulae (5), we can obtain the point estimations of iTE and the four iPSEs in the OR scale.
  (3l) Bootstrap to obtain the standard errors and corresponding 95% confidence intervals. An R package for this analysis can be downloaded free from webpage http://shenglin.blog.nctu.edu.tw/methodology/, or see the Supplementary Material.

**Figure 5:**
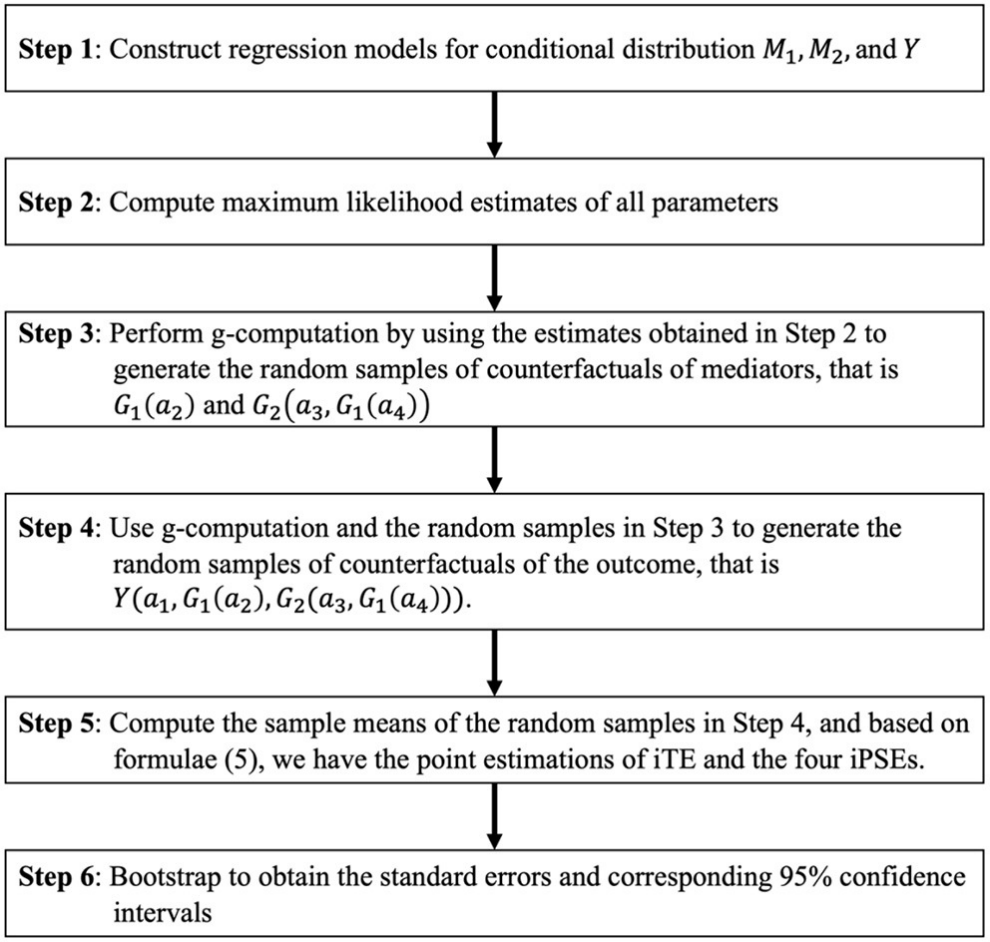
Flow chart for the proposed simulation-based approach. M_1_, M_2_, and Y represent the first mediator, second mediator, and outcome, respectively.

A flow chart for the proposed simulation-based approach is provided in Figure 5. In the approach above, randomly sampling the confounders can be replaced by just using the observed confounders if the sample size is large enough. For a small sample size, the technique of sampling the confounders with a sufficiently large sampling size could improve the stability of the g-computation algorithm approximation. The proposed estimation algorithm in Step 1 demonstrates how to construct regression models for mediators and the outcome with main effects. In practice use, the specifications of these regression models are flexible and are allowed to include any interaction effect. We evaluated the performance of the proposed method *via* a simulation study. The detail of simulation settings is provided in Appendix B, and the result is shown in Section Simulation Study. In Section Simulation Study, we show the operating characteristics of the new proposed estimators and compare them with traditional linear SEM estimators. Additionally, we add mediator interactions into the outcome model for evaluating the characteristics of traditional SEM under model misspecification. We evaluate the two methods by calculating the bias, the empirical standard errors (ESEs), estimated standard errors (SSEs), and coverage rates (COVs). ESE is calculated by the sample standard de*via*tion of estimates over simulations, and SSE is computed by averaging the standard error estimated by bootstrap resampling for each replication. ESEs and SSEs from the bootstrap procedure agree closely for the estimators of iPSEs, implying that the bootstrap procedure provides valid inference. Coverage rate is a proportion of the time that the 95% confidence interval obtained by bootstrap covers the true value of the parameter. In the simulation study, COVs were calculated by using 1,000 replications. If all assumptions we used in the approach are satisfied, COVs should be close to 95%. By contrast, if any assumptions are not met, COVs would be biased.

## Results

### Simulation Study

A simulation study is conducted in Appendix B to show the properties of the proposed estimators and compare them with traditional linear SEM estimators. The corresponding simulation code is provided in Appendix C. Results are shown in Appendix Tables 1 and 2. Without mediator interaction (i.e. θ_*y*, 3_ = 0), both iPSE and SEM methods have small biases. The ESE and SSE values are similar in both methods. iPSE produced slightly larger ESE and SSE values than the SEM method. The coverage rates of both methods are approximately 0.95. When there exists interaction between mediators (i.e. θ_*y*, 3_ = 1, 2, 3), the biases for SEM method increase while the coverage rates approach zero with the exception of *iPSE*_0_ because the SEM estimate for *PSE*_0_ is still unbiased under this scenario. The iPSE method yielded small bias, and the coverage rate was remained approximately 95%.

### Application to Taiwanese REVEAL-HBV Study

The performance of the proposed method was tested in the Taiwanese REVEAL-HBV dataset. Specifically, the method was used to investigate the role of HBV viral load in different time windows as a mediating mechanism in HCV-induced HCC. Here, the outcome was HCC status at the end of follow up, and the exposure of interest was HCV status at enrollment. Mediators M_1_ and M_2_ were HBV viral load at baseline and at follow up, respectively. Baseline confounders included gender, age, smoking status, and ALT level. All analyses were performed in R 3.4.1.

Path-specific effects were estimated using g-computation algorithm (number = 100,000) and bootstrap (resampling size = 1,000). The overall OR of HCV to HCC was 3.122 [95 % CI = (3.108, 3.226)]. For the four paths, the OR of HCV to HCC was 3.910 [95 % CI =(3.785, 4.035)] without mediation by (i.e., without change in) HBV viral load(iPSE_0_) ; 0.873 (95 % CI = (0.853, 0.893) with mediation by baseline but not late HBV viral load (iPSE_1_) ; 0.994 [95 % CI =(0.971, 1.018)] with mediation by late but not baseline HBV viral load (iPSE_2_); and 0.918 [95 % CI = (0.896, 0.941)] with mediation by both baseline and late HBV viral load (iPSE_12_). Note that a high OR for PSE_0_ implies that HBV viral load change conceals the detrimental effect of HCV on HCC. Table 1 lists the above results along with RD and RR scales.

**Table 1:** Total interventional effect of HCV infection on HCC incidence: four interventional path-specific effects with HBV viral load at baseline (M_1_), HBV viral load at follow-up (M_2_) as mediators in scales for risk difference, risk ratio, and odds ratio.

	**Estimate**	**SE**	**95 % CI** ** (lower bound)**	**95% CI** ** (upper bound)**
**Risk difference**				
Total effect	0.096	0.001	0.092	0.099
not *via* M_1_ or M_2_	0.127	0.001	0.123	0.130
*via* M_1_ only	−0.019	0.001	−0.022	−0.015
*via* M_2_ only	0.000	0.001	−0.003	0.002
*via* M_1_ then *via* M_2_	−0.011	0.001	−0.014	−0.007
**Risk ratio**				
Total effect	2.805	0.044	2.718	2.891
not *via* M_1_ or M_2_	3.385	0.050	3.285	3.484
*via* M_1_ only	0.893	0.008	0.876	0.911
*via* M_2_ only	0.995	0.010	0.975	1.015
*via* M_1_ then *via* M_2_	0.930	0.009	0.911	0.950
**Odds ratio**				
Total effect	3.122	0.053	3.018	3.226
not *via* M_1_ or M_2_	3.910	0.063	3.785	4.035
*via* M_1_ only	0.873	0.010	0.853	0.893
*via* M_2_ only	0.994	0.012	0.971	1.018
*via* M_1_ then *via* M_2_	0.918	0.011	0.896	0.941

*HCV, hepatitis C virus; HBV, hepatitis B virus; HCC, hepatocellular carcinoma; SE, standard error; CI, confidence interval*.

## Discussion

Three common approaches to causal mediation analysis include regression-based method, weighting method, and simulation-based method. Since the simulation-based estimation is an approximation of the MLE, it is asymptotically efficient provided all regression models are correctly specified. Contrarily to the regression-based method, the weighting estimation cannot achieve the efficiency bound even if the parametric assumptions for the weights are correct. Here, our approach is more flexible as it allows incorporation of non-linear, polynomial or cross-product interaction terms. Even though OR is the outcome scale of interest here, our method also allows for other non-linear outcome scales.

In some applications, portion mediated (PM) is a measure of interest to assess the proportion of the effect of the exposure mediated by the mediators. On the risk difference scale for continuous outcomes, PM for each mediation path is defined as a ratio of the corresponding iPSE to iTE. For a dichotomous outcome, a odds ratio scale is adopted to define iPSEs, and PMs would be defined on the log odds scale (8). Regardless of on the risk difference scale or the log odds scale, reporting PMs, however, is generally meaningful only if all of iPSEs are in the same direction (e.g., all positive or all negative). As the illustrative example of the Taiwanese REVEAL-HBV dataset, the effects corresponding to the paths involving HBV were negative while other effects were positive. In such a case, PM would not be an appropriate measure to reveal the extent to which mediators affect the causal effect.

There are several noteworthy limitations. Like all simulation-based methods, this approach is computationally intensive. Suppose the time of g-computation algorithm is similar to that of the regression-based method, the computation time would be five-hundred-fold if we constructed confidence intervals by 500 bootstrap repetitions. Note that our approach may be particularly prone to bias due to model misspecification. However, this drawback can be resolved by including quadratic terms for continuous independent variables in regression models and increasing model flexibility. Moreover, the assumptions of no unmeasured confounders may be violated and hard to check. Longitudinal datasets are mostly used to investigate the causal relationship between the exposure and outcome variables. Since mediation analysis or path analysis is usually the secondary analysis of longitudinal datasets, where we mainly focus on exploration of exposure-outcome relationship instead of mediator-outcome, mediator-exposure, or mediator-mediator relationships when collecting confounding variables. We could include application of sensitivity analysis techniques to address violations of these assumptions in future research. Furthermore, estimation of the simulation-based method is unstable when the sample size is small in relation to the complexity of the models, though this is not an issue here because the sample size in Taiwanese HCC cohort is relatively large. It is also worthy to note that a less complicated model is preferred for generating more stable estimations despite flexible model choices in the software.

## Conclusion

HCC ranks sixth in cancer incidence and third in cancer mortality and is a major social burden for all nations (41). Currently, there are about 170 million HCV and 350 million HBV infected cases in the world (42). Our proposed method partially separates the mechanism of HBV and HCV infections on the incidence of HCC. Although HBV and HCV have been confirmed as two etiologic factors for HCC and classified as human carcinogens by the International Agency for Research on Cancer (43), their biological mechanisms remains elusive. Previous studies have shown that HBV and HCV have subadditive interaction on HCC incidence (44–46), and that HCV may suppress the expression and duplication of HBV (47–51). These studies provide evidence that HBV viral activity change may mask the effect of HCV on the HCC risk. In addition, a previous study showed that the early HBV viral activity is an important factor in the development of HCC (15, 16). However, due to the restriction of traditional methods, differentiation of the effects of early HBV viral activity on HCC risk through or not through late HBV viral activity remained difficult. In this study, we utilized the interventional approach to show that both pathways are statistically significant. This result implies that, though the increased HCC caused by HCV infection is not solely through the late HBV viral load (iPSE_2_), both early and late viral load play important roles in the mechanism. Consequently, the decreasing HBV viral load in both time-points can partially prevent the HCC.

Categorical outcomes such as dichotomous or time-to-event outcomes are common, especially epidemiology and health-related fields. Although the iPSE can be identified non-parametrically, the existing regression-based method does not have a closed form (i.e., analytic solution) for non-linear outcome without the rare disease assumption. With our approach, we can ensure that the effect decomposition is applicable for non-linear outcome even without the rare disease assumption. Finally, in our study only allow measurement taken at the end of study as the outcome. It is also important to develop methods for settings with multiple mediators. This can be done by incorporating time-to-event outcome with survival models such as Cox proportional hazard model or accelerated failure time model.

In conclusion, our approach is powerful and versatile for settings with multiple mediators where the traditional PSE is not identified. Furthermore, we facilitate application for mechanism investigation in more complicated settings in epidemiology and health science.

## Data Availability Statement

The data that support the findings of this study are available at http://doi.org/10.1001/jama.295.1.65.

## Author Contributions

A-ST: conceptualization, formal analysis, software, visualization, methodology, and writing—original draft. Y-TH: validation and writing—review and editing. H-IY: data curation, validation, and writing—review and editing. LL: writing—review and editing. S-HL: conceptualization, data curation, funding acquisition, investigation, methodology, project administration, resources, supervision, writing—original draft, and writing—review and editing. All authors contributed to the article and approved the submitted version.

## Funding

A-ST and S-HL are funded by 109-2636-B-009 -001 (Ministry of Science and Technology, Taiwan). Y-TH is funded by AS-CDA-108-M03 (Academia Sinica) and 108-2118-M-001-013-MY5 (Ministry of Science and Technology, Taiwan).

## Conflict of Interest

The authors declare that the research was conducted in the absence of any commercial or financial relationships that could be construed as a potential conflict of interest.

## Publisher's Note

All claims expressed in this article are solely those of the authors and do not necessarily represent those of their affiliated organizations, or those of the publisher, the editors and the reviewers. Any product that may be evaluated in this article, or claim that may be made by its manufacturer, is not guaranteed or endorsed by the publisher.

## References

[1] MacKinnon DP. Introduction to statistical mediation analysis. New York, NY: Routledge. (2008).

[2] Pearl J. Causal inference in statistics: An overview. Stat Surv. (2009) 3:96–146. 10.1214/09-SS05718320210

[3] Robins J. A new approach to causal inference in mortality studies with a sustained exposure period—application to control of the healthy worker survivor effect. Mathematical Modelling. (1986) 7:1393–512. 10.1016/0270-0255(86)90088-629883322

[4] Rubin DB. Formal mode of statistical inference for causal effects. J Stat Plan Inference. (1990) 25:279–92. 10.1016/0378-3758(90)90077-8

[5] Robins JM Greenland S. Identifiability and exchangeability for direct and indirect effects. Epidemiology. (1992) 143–l55. 10.1097/00001648-199203000-00013 1576220

[6] Pearl J. Direct and indirect effects. Proceedings of the Seventeenth conference on Uncertainty in artificial intelligence. San Francisco, CA, USA: Morgan kaufmann publishers Inc. (2001) p. 411–420.

[7] VanderWeele T. Explanation in Causal Inference: Methods for Mediation and Interaction. New York, NY: Oxford University Press. (2015).

[8] VanderWeele TJ Vansteelandt S. Odds ratios for mediation analysis for a dichotomous outcome. Am J Epidemiol. (2010) 172:1339–48. 10.1093/aje/kwq332 21036955 PMC2998205

[9] Lange T Hansen JV. Direct and indirect effects in a survival context. Epidemiology. (2011) 22:575–81. 10.1097/EDE.0b013e31821c680c 21552129

[10] Martinussen T Vansteelandt S Gerster M. Hjelmborg JvB. Estimation of direct effects for survival data by using the Aalen additive hazards model. J Royal Stat Soc. (2011) 73:773–88. 10.1111/j.1467-9868.2011.00782.x

[11] Tchetgen Tchetgen EJ. On causal mediation analysis with a survival outcome. Int J Biostat. (2011) 7:1–38. 10.2202/1557-4679.1351 22049268 PMC3204669

[12] VanderWeele TJ. Causal mediation analysis with survival data. Epidemiology (Cambridge, Mass). (2011) 22:582. 10.1097/EDE.0b013e31821db37e 21642779 PMC3109321

[13] Valeri L VanderWeele TJ. Mediation analysis allowing for exposure–mediator interactions and causal interpretation: Theoretical assumptions and implementation with SAS and SPSS macros. Psychol Methods. (2013) 18:137. 10.1037/a0031034 23379553 PMC3659198

[14] Lin SH Young J Logan R Tchetgen Tchetgen EJ VanderWeele TJ. Parametric mediational g-formula approach to mediation analysis with time-varying exposures, mediators, and confounders. Epidemiology. (2017) 28:266–74. 10.1097/EDE.0000000000000609 27984420 PMC5285457

[15] Huang Y-T Yang H-I. Causal mediation analysis of survival outcome with multiple mediators. Epidemiology. (2017) 28:370–8. 10.1097/EDE.0000000000000651 28296661 PMC5408128

[16] Huang Y-T Yang H-I Liu J Lee M-H Freeman JR Chen C-J. Mediation analysis of hepatitis B and C in relation to hepatocellular carcinoma risk. Epidemiology. (2016) 27:14–20. 10.1097/EDE.0000000000000390 26443934

[17] Taguri M Featherstone J Cheng J. Causal mediation analysis with multiple causally non-ordered mediators. Stat Methods Med Res. (2015) 27:3–19. 10.1177/0962280215615899 26596350 PMC5698181

[18] VanderWeele TJ Vansteelandt S. Mediation analysis with multiple mediators. Epidemiol Method. (2014) 2:95–115. 10.1515/em-2012-0010 25580377 PMC4287269

[19] Avin C Shpitser I Pearl J. Identifiability of path-specific effects. Los Angeles, CA: Department of Statistics, UCLA. (2005).

[20] VanderWeele TJ Vansteelandt S Robins JM. Effect decomposition in the presence of an exposure-induced mediator-outcome confounder. Epidemiology. (2014) 25:300–6. 10.1097/EDE.0000000000000034 24487213 PMC4214081

[21] Daniel R De Stavola B Cousens S Vansteelandt S. Causal mediation analysis with multiple mediators. Biometrics. (2015) 71:1–14. 10.1111/biom.12248 25351114 PMC4402024

[22] Huang YT Cai T. Mediation analysis for survival data using semiparametric probit models. Biometrics. (2015). 10.1111/biom.12445 26618735

[23] Huang Y-T Yang H-I Liu J Lee M-H Freeman JR Chen C-J. Mediation analysis of hepatitis b and c in relation to hepatocellular carcinoma risk. Epidemiology (Cambridge, Mass). (2015) 27:14–20. 26443934 10.1097/EDE.0000000000000390

[24] VanderWeele TJ. Bias formulas for sensitivity analysis for direct and indirect effects. Epidemiology (Cambridge, Mass). (2010) 21:540. 10.1097/EDE.0b013e3181df191c 20479643 PMC4231822

[25] VanderWeele TJ. Unmeasured confounding and hazard scales: sensitivity analysis for total, direct and indirect effects. Eur J Epidemiol. (2013) 28:113–7. 10.1007/s10654-013-9770-6 23371044 PMC3606287

[26] Lin S-H VanderWeele T. Interventional approach for path-specific effects. J Causal Inference. (2017) 5. 10.1515/jci-2015-0027

[27] Vansteelandt S Daniel RM. Interventional effects for mediation analysis with multiple mediators. Epidemiology (Cambridge, Mass). (2017) 28:258. 10.1097/EDE.0000000000000596 27922534 PMC5289540

[28] Imai K Keele L Tingley D. A general approach to causal mediation analysis. Psychol Methods. (2010) 15:309. 10.1037/a0020761 20954780

[29] Imai K Keele L Tingley D Yamamoto T. Causal mediation analysis using R. Advances in social science research using R. Springer. (2010). p. 196:129–154. 10.1007/978-1-4419-1764-5_8

[30] Tingley D Yamamoto T Hirose K Keele L Imai K. Mediation: R package for causal mediation analysis. J Stat Softw. (2014) 59:1–38. 10.18637/jss.v059.i0526917999

[31] Wang A Arah OA. G-computation demonstration in causal mediation analysis. Eur J Epidemiol. (2015) 30:1119–27. 10.1007/s10654-015-0100-z 26537707 PMC4674449

[32] Taubman SL Robins JM Mittleman MA Hernán MA. Intervening on risk factors for coronary heart disease: an application of the parametric g-formula. Int J Epidemiol. (2009) 38:1599–611. 10.1093/ije/dyp192 19389875 PMC2786249

[33] Hernan JMRaMA. Estimation of the causal effects of time-varying exposures. In: Fitzmaurice G Verbeke G Molenberghs G editor. Longitudinal Data Analysis. Boca Raton, FL: Chapman & Hall/CRC. (2009).

[34] Westreich D CS Young JG Palella F Tien PC Kingsley L Gange SJ . The parametric g-formula to estimate the effect of highly active antiretroviral therapy on incident AIDS or death. Stat Med. (2012). 10.1002/sim.5316 22495733 PMC3641816

[35] Lin SH Young JG Logan R VanderWeele TJ. Mediation analysis for a survival outcome with time-varying exposures, mediators, and confounders. Stat Med. (2017) 36:4153–66. 10.1002/sim.7426 28809051 PMC6242332

[36] Chen C-J Yang H-I Su J Jen C-L You S-L Lu S-N . Risk of hepatocellular carcinoma across a biological gradient of serum hepatitis B virus DNA level. JAMA. (2006) 295:65–73. 10.1001/jama.295.1.65 16391218

[37] Chen CL Yang HI Yang WS Liu CJ Chen PJ You SL . Metabolic factors and risk of hepatocellular carcinoma by chronic hepatitis B/C infection: a follow-up study in Taiwan. Gastroenterology. (2008) 135:111–21. 10.1053/j.gastro.2008.03.073 18505690

[38] Iloeje UH Yang HI Jen CL Su J Wang LY You SL . Risk and predictors of mortality associated with chronic hepatitis B infection. Clin Gastroenterol Hepatol. (2007) 5:921–31. 10.1016/j.cgh.2007.06.015 17678844

[39] Lee M-H Yang H-I Lu S-N Jen C-L Yeh S-H Liu C-J . Hepatitis C virus seromarkers and subsequent risk of hepatocellular carcinoma: long-term predictors from a community-based cohort study. Journal of Clinical Oncology. (2010) 28:4587–93. 10.1200/JCO.2010.29.1500 20855826

[40] Hernán M. A definition of causal effect for epidemiological research. J Epidemiol Community Health. (2004) 58:265–71. 10.1136/jech.2002.006361 15026432 PMC1732737

[41] Parkin DM Bray F Ferlay J Pisani P. Global cancer statistics, 2002. CA Cancer J Clin. (2005) 55:74–108. 10.3322/canjclin.55.2.74 15761078

[42] Lauer GM Walker BD. Hepatitis C virus infection. N Engl J Med. (2001) 345:41–52. 10.1056/NEJM200107053450107 11439948

[43] WHO. IARC Working Group on the Evaluation of Carcinogenic Risks to Humans. Vol. 59. International Agency for Research on Cancer, & World Health Organization. (1994). Available online at: https://monographs.iarc.who.int/wp-content/uploads/2018/06/mono93.pdf

[44] Huang Y-T Jen C-L Yang H-I Lee M-H Su J Lu S-N . Lifetime risk and sex difference of hepatocellular carcinoma among patients with chronic hepatitis B and C. J Clin Oncol. (2011) 29:3643–50. 10.1200/JCO.2011.36.2335 21859997 PMC4874144

[45] Kuper H Tzonou A Kaklamani E Hadziyannis S Tasopoulos N Lagiou P . Hepatitis B and C viruses in the etiology of hepatocellular carcinoma; a study in Greece using third-generation assays. Cancer Causes Control. (2000) 11:171–5. 10.1023/A:1008951901148 10710202

[46] Sun C-A Wu D-M Lin C-C Lu S-N You S-L Wang L-Y . Incidence and cofactors of hepatitis C virus-related hepatocellular carcinoma: a prospective study of 12,008 men in Taiwan. Am J Epidemiol. (2003) 157:674–82. 10.1093/aje/kwg041 12697571

[47] Tsiquaye K Tovey G Kessler H Hu S Lu XZ Zuckerman A . Non-A, non-b hepatitis in persistent carriers of hepatitis b virus. J Med Virol. (1983) 11:179–89. 10.1002/jmv.1890110302 6408222

[48] Liaw YF. Role of hepatitis C virus in dual and triple hepatitis virus infection. Hepatology. (1995) 22:1101–8. 10.1002/hep.1840220413 7557857

[49] Koike K Yotsuyanagi H Moriya K Kurokawa K Yasuda K Lino S . Dominant replication of either virus in dual infection with hepatitis viruses B and C. J Med Virol. (1995) 45:236–9. 10.1002/jmv.1890450222 7539830

[50] Shih CM Lo SJ Miyamura T Chen SY Lee Y. Suppression of hepatitis B virus expression and replication by hepatitis C virus core protein in HuH-7 cells. J Virol. (1993) 67:5823–32. 10.1128/jvi.67.10.5823-5832.1993 8396658 PMC238000

[51] Schüttler CG Fiedler N Schmidt K Repp R Gerlich WH Schaefer S. Suppression of hepatitis B virus enhancer 1 and 2 by hepatitis C virus core protein. J Hepatol. (2002) 37:855–62. 10.1016/S0168-8278(02)00296-9 12445429

