# Supplementary material for: G-Computation to Causal Mediation Analysis With Sequential Multiple Mediators—Investigating the Vulnerable Time Window of HBV Activity for the Mechanism of HCV Induced Hepatocellular Carcinoma
Source: Front Public Health. 2022 Jan 7;9:757942. doi: 10.3389/fpubh.2021.757942 (PMC8779208; doi:10.3389/fpubh.2021.757942)
Supplement: Supplementary file 1 [file Presentation_1.pdf]

Appendix:
G-computation to causal mediation analysis with sequential multiple mediators – investigating the vulnerable time window of HBV activity for the mechanism of HCV induced hepatocellular carcinoma

An-Shun Tai[1], Yen-Tsung Huang[2], Hwai-I Yang[3], Lauren V. Lan[4,1], Sheng-Hsuan Lin[1*]

[1] Institute of Statistics, National Yang Ming Chiao Tung University, Hsinchu, Taiwan
1001 University Road, Hsinchu 300, Taiwan
[2] Institute of Statistical Science, Academia Sinica, Taipei, Taiwan
128 Academia Road, Section 2, Nankang, Taipei 115, Taiwan
[3] Genomics Research Center, Academia Sinica, Taipei, Taiwan
128 Academia Road, Section 2, Nankang, Taipei, 115, Taiwan
[4] Department of Biostatistics, Johns Hopkins University, Baltimore, MD, USA
615 N. Wolfe Street, Baltimore, MD 21205, US

Appendix A: Definition of iPSE in RD and RR scales

Formulas (3) to (5) can be expressed in RD scale as follows:

$$\begin{aligned} RD_{iPSE0} &= \Psi(1,0,0,0)-\Psi(0,0,0,0) \\ RD_{iPSE1} &= \Psi(1,1,0,0)-\Psi(1,0,0,0) \\ RD_{iPSE2} &= \Psi(1,1,1,0)-\Psi(1,1,0,0) \\ RD_{iPSE12} &= \Psi(1,1,1,1)-\Psi(1,1,1,0) \end{aligned} \qquad (3.RD)$$

where $\Psi(a_1,a_2,a_3,a_4)$ is defined as $Pr(Y(a_1,G_1(a_2),G_2(a_3,G_1(a_4)))=1)$.

$$RD_{iTE} = RD_{iPSE0}+RD_{iPSE1}+RD_{iPSE2}+RD_{iPSE12} \qquad (4.RD)$$

All RDs can be identified in terms of Q under four assumptions of no unmeasured confounders :

$$\begin{aligned} RD_{iTE} &= Q(1,1,1,1)-Q(0,0,0,0) \\ RD_{iPSE0} &= Q(1,0,0,0)-Q(0,0,0,0) \\ RD_{iPSE1} &= Q(1,1,0,0)-Q(1,0,0,0) \\ RD_{iPSE2} &= Q(1,1,1,0)-Q(1,1,0,0) \end{aligned} \qquad (5.RD)$$

RD_{iPSE12} = Q(1,1,1,1)-Q(1,1,1,0)

Similarly, regarding RR scales, these formulas can be expressed in terms of RR as follows:

RR_{iPSE0} = Ψ(1,0,0,0)/Ψ(0,0,0,0)
RR_{iPSE1} = Ψ(1,1,0,0)/Ψ(1,0,0,0)
RR_{iPSE2} = Ψ(1,1,1,0)/Ψ(1,1,0,0)
RR_{iPSE12} = Ψ(1,1,1,1)/Ψ(1,1,1,0) (3.RR)

RR_{iTE} = RR_{iPSE0}×RR_{iPSE1}×RR_{iPSE2}×RR_{iPSE12} (4.RR)

All RRs can be identified in terms of Q under four assumptions of no unmeasured confounders:

RR_{iTE} = Q(1,1,1,1)/Q(0,0,0,0)
RR_{iPSE0} = Q(1,0,0,0)/Q(0,0,0,0)
RR_{iPSE1} = Q(1,1,0,0)/Q(1,0,0,0)
RR_{iPSE2} = Q(1,1,1,0)/Q(1,1,0,0)
RR_{iPSE12} = Q(1,1,1,1)/Q(1,1,1,0) (5.RR)

Appendix B: Simulation setting

A simulation study was performed to observe the operating characteristics of the proposed estimators and to compare them with conventional linear SEM estimators. Since the closed form for parameters $Y, M_1,$ and M_2 requires the assumption of linear models, (M_1, M_2, Y) are all assumed to be continuous.

For simulated data with sample size 1,000 and 5,000, we generated the three baseline confounders (C_1, C_2, C_3), one exposure (A), two mediators (M_1, M_2) and outcome (Y), as follows:

$C_1 \sim normal(0, 1)$
$C_2 \sim normal(0, 1)$

$C_3 \sim normal(0, 1)$
$A \sim \text{bernoulli}(0.5)$
$M_1 = 1 + A + C_1 + C_2 + C_3 + \varepsilon_1,\ \varepsilon_1 \sim normal(0, 1)$
$M_2 = 1 + A + M_1 + C_1 + C_2 + C_3 + \varepsilon_2,\ \varepsilon_2 \sim normal(0, 1)$
$Y = 1 + A + M_1 + M_2 + \theta_{y,3} M_1 M_2 + C_1 + C_2 + C_3 + \varepsilon_y,\ \varepsilon_y \sim normal(0, 1)$

Coefficients for mediator interactions $\theta_{y,3}$ were set to 0, 1, 2, and 3 to evaluate the characteristics of conventional SEM under model misspecification. Simulations were performed using g-computation algorithm (sample size = $1{,}000$) and bootstrap analysis (resampling size = $1{,}000$). Traditional SEM was also fitted for comparison. The simulation was repeated 1,000 times using the above procedure. Bias, empirical standard errors (ESEs), estimated standard errors (SSEs), and coverage rates (COVs) were calculated for both iPSE and SEM methods.

Appendix C: Simulation code

```
n1=1000
n2=200
boot=1000 #bootstraping number
nn=1000 #g computation number

data_generation=function(n1,interaction){
   c1=rnorm(n1,0,1)
   c2=rnorm(n1,0,1)
   c3=rnorm(n1,0,1)
   a=rbinom(n1,1,0.5)
   m1=rnorm(n1,1+a+c1+c2+c3,1)
   m2=rnorm(n1,1+a+c1+c2+c3+m1,1)
   y=rnorm(n1,1+a+c1+c2+c3+m1+m2+(interaction*(m1*m2)),1)
   data1=cbind(c1,c2,c3,a,m1,m2,y)
   return(data1)
}
#tt=replicate(n2,data_generation(n1,0),simplify = "array")
#data=data_generation(n1,0)

pse=function(data,nn){
   data=as.data.frame(data)
   m1_model=lm(m1~a+c1+c2+c3,data)
   m2_model=lm(m2~m1+a+c1+c2+c3,data)
   y_model=lm(y~m2+m1+a+c1+c2+c3+m1*m2,data)
   c1d=sample(data$c1,nn,replace = TRUE)
   c2d=sample(data$c2,nn,replace = TRUE)
   c3d=sample(data$c3,nn,replace = TRUE)

   #m1
   ma=predict(m1_model,data.frame(a=1,c1=c1d
                                  ,c2=c2d
                                  ,c3=c3d))
```

```
#m0
mb=predict(m1_model,data.frame(a=0,c1=c1d
                                ,c2=c2d
                                ,c3=c3d))

#m(1,m(1))
mc=predict(m2_model,data.frame(m1=ma,a=1
                                ,c1=c1d
                                ,c2=c2d
                                ,c3=c3d))

#m(1,m(0))
md=predict(m2_model,data.frame(m1=mb,a=1
                                ,c1=c1d
                                ,c2=c2d
                                ,c3=c3d))
#m(0,m(0))
me=predict(m2_model,data.frame(m1=mb,a=0
                                ,c1=c1d
                                ,c2=c2d
                                ,c3=c3d))


##
#y(1,m(1),m(1,m(1)))
mf=predict(y_model,data.frame(m2=mc
                               ,m1=ma,a=1
                               ,c1=c1d
                               ,c2=c2d
                               ,c3=c3d))
#y(1,m(1),m(1,m(0)))
mg=predict(y_model,data.frame(m2=md
                               ,m1=ma,a=1
                               ,c1=c1d
                               ,c2=c2d
```

```
                                          ,c3=c3d))

  #y(1,m(1),m(0,m(0)))

  mh=predict(y_model,data.frame(m2=me
                                          ,m1=ma,a=1
                                          ,c1=c1d
                                          ,c2=c2d
                                          ,c3=c3d))

  #y(1,m(0),m(0,m(0)))
  mi=predict(y_model,data.frame(m2=me
                                          ,m1=mb,a=1
                                          ,c1=c1d
                                          ,c2=c2d
                                          ,c3=c3d))

  #y(0,m(0),m(0,m(0)))
  mj=predict(y_model,data.frame(m2=me
                                          ,m1=mb,a=0
                                          ,c1=c1d
                                          ,c2=c2d
                                          ,c3=c3d))



  pse12=mean(mf)-mean(mg)#pse12
  pse2=mean(mg)-mean(mh)#pse2
  pse1=mean(mh)-mean(mi)#pse1
  pse0=mean(mi)-mean(mj) #pse0
  return(c(pse0,pse1,pse2,pse12))


}
bootstrap=function(data){
  r=sample(1:n1,n1,replace=TRUE)
```

```
  ndata=data[r,]
  return(ndata)
}

tt=replicate(n2,data_generation(n1,0),simplify = "array")
result=array(data = NA,dim=c(4,boot,n2))
for(i in 1:n2){
  result[,,i]=replicate(boot,pse(bootstrap(tt[,,i]),nn))
}

xx=apply(tt,3,function(x){
  pse(x,nn)
}
)

meandata=apply(result,3,rowMeans)
sddata=apply(result,3,function(x){
  apply(x,1,sd)
})


sse1=apply(meandata,1,sd)
ese1=apply(sddata,1,mean)
tee=(1>(meandata-1.96*sddata) & 1<(meandata+1.96*sddata))
covrate1=apply(tee, 1, mean)
mmean1=apply(xx,1,mean)
```

Appendix Table 1. Sample size 1000 for simulation-based method and structural equation model.

			G-computation approach for counterfactual model analysis				Linear Structural Equation Model			
$\theta_{y,3}$	Parameters	True Value	Bias	ESE	SSE	COV	Bias	ESE	SSE	COV
0	$iPSE_0$	1	-0.009	0.194	0.195	0.953	0.000	0.080	0.078	0.933
	$iPSE_1$	1	0.006	0.218	0.215	0.945	0.001	0.078	0.078	0.950
	$iPSE_2$	1	0.001	0.227	0.232	0.957	-0.001	0.075	0.078	0.955
	$iPSE_{12}$	1	-0.010	0.250	0.249	0.953	-0.005	0.079	0.077	0.937
1	$iPSE_0$	1	-0.004	0.468	0.463	0.943	0.000	0.079	0.078	0.950
	$iPSE_1$	3	-0.011	0.607	0.582	0.942	-1.999	0.077	0.078	0.000
	$iPSE_2$	3	0.026	0.742	0.716	0.940	-2.001	0.077	0.078	0.000
	$iPSE_{12}$	3	0.019	0.851	0.828	0.940	-1.999	0.078	0.078	0.000
2	$iPSE_0$	1	0.000	0.793	0.784	0.946	0.001	0.079	0.078	0.938
	$iPSE_1$	5	-0.017	1.035	0.992	0.939	-4.001	0.078	0.078	0.000
	$iPSE_2$	5	-0.015	1.239	1.229	0.943	-3.996	0.080	0.078	0.000
	$iPSE_{12}$	5	-0.023	1.472	1.433	0.931	-4.002	0.075	0.078	0.000
3	$iPSE_0$	1	-0.032	1.109	1.113	0.939	-0.002	0.076	0.078	0.947
	$iPSE_1$	7	0.099	1.510	1.410	0.934	-5.996	0.081	0.078	0.000
	$iPSE_2$	7	-0.038	1.651	1.751	0.971	-6.003	0.078	0.078	0.000
	$iPSE_{12}$	7	0.059	2.100	2.050	0.947	-5.995	0.078	0.078	0.000

$\theta_{y,3}$: mediator interaction term; ESE: empirical standard error; SSE: estimated standard error; COV: coverage rate.

Appendix Table 2. Sample size 5000 for simulation-based method and structural equation model.

			G-computation approach for counterfactual model analysis				Linear Structural Equation Model			
$\theta_{y,3}$	Parameters	True Value	Bias	ESE	SSE	COV	Bias	ESE	SSE	COV
0	$iPSE_0$	1	-0.002	0.186	0.182	0.942	0.002	0.034	0.035	0.962
	$iPSE_1$	1	0.000	0.202	0.203	0.950	0.000	0.035	0.035	0.957
	$iPSE_2$	1	0.000	0.216	0.222	0.959	0.001	0.034	0.035	0.959
	$iPSE_{12}$	1	-0.001	0.246	0.239	0.947	0.000	0.034	0.035	0.964
1	$iPSE_0$	1	-0.013	0.446	0.457	0.959	0.001	0.036	0.035	0.936
	$iPSE_1$	3	0.008	0.550	0.560	0.944	-2.000	0.034	0.035	0.000
	$iPSE_2$	3	-0.022	0.705	0.688	0.949	-2.001	0.034	0.035	0.000
	$iPSE_{12}$	3	0.002	0.795	0.797	0.946	-2.000	0.035	0.035	0.000
2	$iPSE_0$	1	0.037	0.776	0.780	0.949	0.000	0.034	0.035	0.958
	$iPSE_1$	5	-0.104	0.953	0.959	0.948	-3.999	0.035	0.035	0.000
	$iPSE_2$	5	0.039	1.159	1.184	0.948	-4.002	0.034	0.035	0.000
	$iPSE_{12}$	5	-0.022	1.411	1.382	0.940	-4.000	0.034	0.035	0.000
3	$iPSE_0$	1	-0.018	1.071	1.110	0.965	-0.002	0.034	0.035	0.948
	$iPSE_1$	7	-0.042	1.323	1.362	0.956	-5.999	0.035	0.035	0.000
	$iPSE_2$	7	0.020	1.605	1.683	0.961	-6.000	0.036	0.035	0.000
	$iPSE_{12}$	7	-0.029	1.970	1.973	0.948	-5.999	0.034	0.035	0.000

$\theta_{y,3}$: mediator interaction term; ESE: empirical standard error; SSE: estimated standard error; COV: coverage rate.
